# Transcriptional Analysis of Drought-Induced Genes in the Roots of a Tolerant Genotype of the Common Bean (*Phaseolus vulgaris* L.)

**DOI:** 10.3390/ijms14047155

**Published:** 2013-03-28

**Authors:** Gustavo Henrique Recchia, Danielle Gregorio Gomes Caldas, Ana Luiza Ahern Beraldo, Márcio José da Silva, Siu Mui Tsai

**Affiliations:** 1 Center of Nuclear Energy for Agriculture, Laboratory of Cellular and Molecular Biology, University of São Paulo, P.O. Box 96, Piracicaba 13400-970, SP, Brazil; E-Mails: ghrecchia@cena.usp.br (G.H.R.); alberaldo@gmail.com (A.L.A.B.); tsai@cena.usp.br (S.M.T.); 2 Center for Molecular Biology and Genetic Engineering, Center of Plant Molecular Biology, University of Campinas, Campinas 13083-875, SP, Brazil; E-Mail: marciojs@unicamp.br

**Keywords:** *Phaseolus vulgaris* L., drought stress, root, suppressive subtractive hybridization library, gene expression

## Abstract

In Brazil, common bean (*Phaseolus vulgaris* L.) productivity is severely affected by drought stress due to low technology cultivation systems. Our purpose was to identify differentially expressed genes in roots of a genotype tolerant to water deficit (BAT 477) when submitted to an interruption of irrigation during its development. A SSH library was constructed taking as “driver” the genotype Carioca 80SH (susceptible to drought). After clustering and data mining, 1572 valid reads were obtained, resulting in 1120 ESTs (expressed sequence tags). We found sequences for transcription factors, carbohydrates metabolism, proline-rich proteins, aquaporins, chaperones and ubiquitins, all of them organized according to their biological processes. Our suppressive subtractive hybridization (SSH) library was validated through RT-qPCR experiment by assessing the expression patterns of 10 selected genes in both genotypes under stressed and control conditions. Finally, the expression patterns of 31 ESTs, putatively related to drought responses, were analyzed in a time-course experiment. Our results confirmed that such genes are more expressed in the tolerant genotype during stress; however, they are not exclusive, since different levels of these transcripts were also detected in the susceptible genotype. In addition, we observed a fluctuation in gene regulation over time for both the genotypes, which seem to adopt and adapt different strategies in order to develop tolerance against this stress.

## 1. Introduction

Plants are frequently exposed to stress-inducing environmental conditions, such as drought or floods, intense heat or cold, excessive soil salinity, insufficient availability of nutrients, changes in lightness, and the presence of heavy metals, that affect their growth, development and productivity [1]. The tolerance or susceptibility of a species to abiotic stress depends on several factors, with the genotype and the phenological stage of development being the most important [2].

The common bean (*P. vulgaris* L.) is the second most important legume crop in the world [3]. This culture is considered the central figure in the daily diet of more than 300 million people around the world [4], and is characterized as the main source of protein for populations in Latin America and East Africa [5].

The conditions under which this crop is grown are extremely variable [6]. While its production tends to be centered on small areas, the planting system used can vary from widely mechanized, irrigated and intensive production [7], to complexes of small farmers who rely solely on rainwater for the irrigation of the fields. It is estimated that over 73% of total production in Latin America and 40% of the total in Africa occurs in micro-climatic conditions which face moderate to severe water deficit at some point during their cultivation [8]. This leads to a reduction in biomass and number of seeds per pod, while affecting days to reach maturation, harvesting rates, production and seed weight and nitrogen fixation [9].

Abiotic stresses trigger a wide range of responses in the plant, from changes in patterns of gene expression and cellular metabolism to changes in growth and yield; the length and severity with which a stress is imposed provides the greatest influence on the plant response [2]. Several studies have hypothesized that the main ability of a plant to avoid drought stress is to change its roots distribution in the soil [10,11]. Therefore, it is extremely important to understand how genotypes considered to be tolerant respond to stress in order to select genes that might be useful to establish programs of genetic improvement for crops important to human consumption [7].

Torres *et al.* (2006) [12] used DDRT (differential display RT-qPCR) and identified 16 *P. vulgaris* clones related to pre-regulation of stress response in roots under water deficit, allowing the identification of four genes. Their involvement in signaling, protein structural changes, translocations, chaperonin and modulation of root growth were also observed.

Root cDNA libraries were differentially screened to isolate water deficit-responsive transcripts in the relatively drought-resistant plant tepary bean (*Phaseolus acutifolius*) [13]. A novel bZIP transcription factor was identified and showed to accumulate also in roots of *P. vulgaris* in response to water deficit.

Through the construction of suppressive subtractive hybridization (SSH) cDNA libraries contrasting tolerant (Pinto Villa) and susceptible (Carioca) varieties of common bean, Montalvo-Henández *et al*. (2008) [14] were able to identify a new aquaporin. Under drought stress, it was evidenced that levels of mRNAs for known aquaporins decay to levels unable to be detected in the susceptible cultivar. On the other hand, transcript levels for these same aquaporins were stable in the phloem tissue of the tolerant variety.

SSH method has been successfully used to construct cDNA libraries enriched in transcripts that are differentially expressed in target tissues, developmental stages, and specific treatments in various biological systems [15] and has been employed for different purposes, such as fungal infection monitoring in citrus [16], tolerance to water deficit in pigeon pea [17], flower development of orange plants [18], tolerance to Varroa destructor mite in honey bees [19].

The main objective of this work was to identify differentially expressed genes of both roots under drought and of tolerant and susceptible common bean genotypes in order to better understand their molecular mechanisms and to draw a model for drought response in roots. A novel collection of *P. vulgaris* EST sequences was generated constituting an important step in finding candidate genes to improve common bean response to drought.

## 2. Results and Discussion

### 2.1. Soil Water Moisture

**Figure 1:**
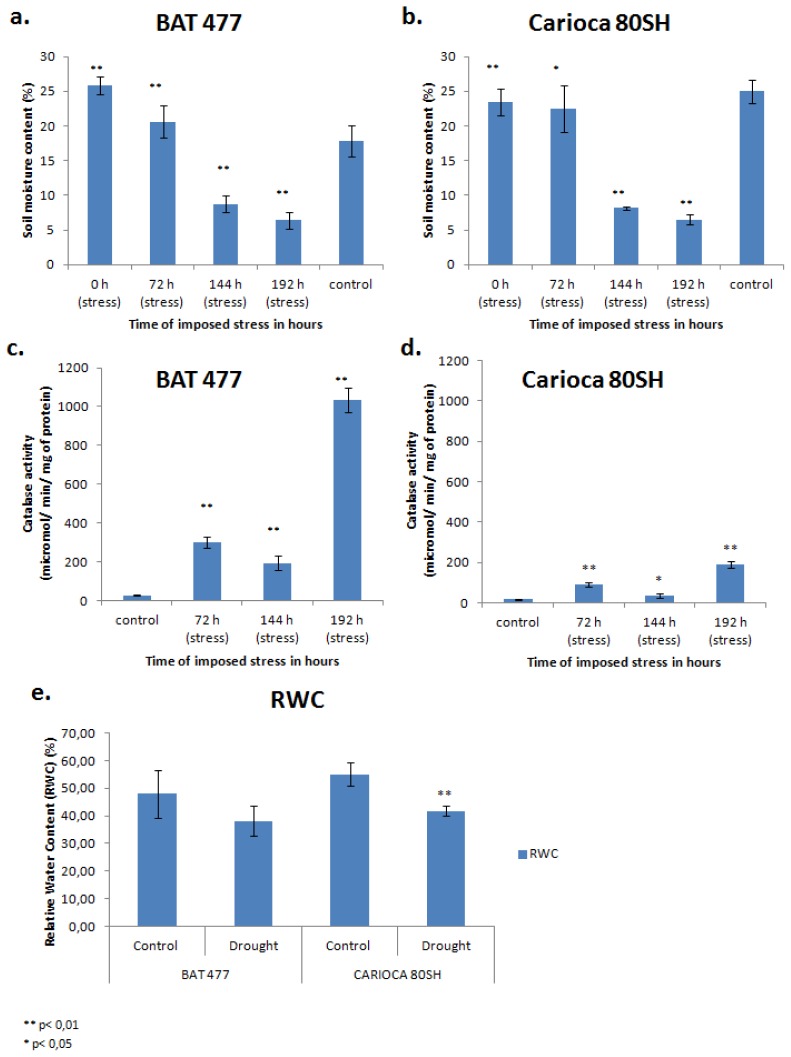
Average soil moisture content (%) obtained from pots cultivated with (**a**) BAT 477 and (**b**) Carioca 80SH common bean genotypes. Average enzymatic activity of catalase (μmol^−1^ mg^−1^ protein) inferred from leaf samples harvested from (**c**) BAT 477 and (**d**) Carioca 80SH. The measurements were taken from soil samples and leaves collected after 0, 72, 144 and 196 h of drought stress, along with their respective well-watered controls, which represents an average of the whole period; (**e**) Relative Water Content (%) of leaves harvested after 6 h of 10% PEG (polyethylene glycol) treatment. Experiments were conducted in five biological replicates.

In order to monitor drought imposition to the plants, soil moisture content was measured during the experiment. Four samplings were taken; the first at the time of stress imposition (0 h), and the next three after 72, 144 and 192 h. A positive correlation in soil water availability can be seen as the soil moisture for both stress treatments decreases gradually after the beginning of water deprivation (Figure 1a,b). Soil moisture content of the controls was also obtained and variations between both genotypes (Figure 1a,b) could mark the differences in their root system architecture.

In field studies, where similar parameters were adopted, it was detected that the wilting point of common bean was attained after the soil moisture reaches 16.6% [20]. However, in our study, this critical condition was not detected, since after 192 h of water suspension, where the soil moisture for BAT 477 was 6.4% and for Carioca 80SH was 6.5%, we started a rehydration of drought-stressed plants and they were all able to recover. Hence, we chose a sampling with soil moisture between 16% and 6% to be studied here.

### 2.2. Biological Effects of Drought on Plants

One of the direct consequences of drought stress is the triggering of another usual type of abiotic stress, the oxidative stress. Such a situation leads to the release of the reactive oxygen species (ROS), which are formed during redox reactions during the incomplete reduction of oxygen and/or the oxidation of water through the electron transporting chains in mitochondrias and chloroplasts [2]. Compounds, such as the hydrogen peroxide, cause extensive cellular damages and can lead to photosynthesis inhibition [21].

Detoxification enzymes such as catalase, ascorbate peroxidase, superoxide dismutase and glutathione *S*-transferase, present a preponderant role in plant protection against damages caused by ROS. These enzymes are coded by a group of genes with role in cell protection against dehydration effects [22]. Thereby, when plants are submitted to abiotic stress, an increase on these enzymes’ activity may be expected.

We analyzed the enzymatic activity of catalase in leaves of common bean. In a study comparing three different genotypes of chickpea (*Cicer arietinum* L.), the catalase enzymatic activity assay in leaves was very efficient and able to distinguish the tolerant genotypes from those susceptible to drought. For the three investigated stages (seedling, podding and flowering stages), an increase in catalase activity in the tolerant genotype (MCC877) was verified in relation to the susceptible (MCC68 and MCC448) [23].

As can be inferred by Figure 1c,d, the expected increase in catalase activity in our leaves was achieved for both genotypes during the days of treatment, confirming the physiological state of the plants due to the drought. This increase in catalase activity was around three times higher in leaves of BAT 477 than in Carioca 80SH for the first 72 h of water deficit, and, by the end of the experiment (after 192 h), 5.5 times higher. These results may also represent a plausibly important differentiation in the drought-stress response between genotypes, since the fold change in catalase activity for Carioca 80SH was 200, while BAT 477 showed a 1100 fold change.

Another interesting pattern that is found in Figure 1c,d is that, for both the genotypes, a decrease in catalase activity occurs after 144 h of stress to levels lower than those detected in the first 72 h, followed by an intense recovery during the last hours of the experiment. Further investigations must be taken for elucidating these results, but this could be a reflection of a time-scale shift in the mechanisms by which the plant endures the stress period.

Further investigation to accomplish a better characterization of the drought response of both the genotypes was carried out. Plants were treated with a 10% polyethylene glycol (PEG) solution; leaves were harvested and “fresh,” “turgid” and “dry” weights were measured to obtain the percentage of relative water content (RWC). Averages of RWC (%) can be visualized in Figure 1e. A profound reduction in RWC (%) was observed for both the genotypes during drought stress. However, this was only statistically significant for Carioca 80SH, confirming previous studies that indicated this genotype as susceptible to drought.

All data shown in Figure 1 confirm the success in imposing drought stress to the plants of the two genotypes in our experiment, as well as genotype-specific responses.

### 2.3. Functional Annotation and Classification of Transcripts

To identify genes differentially expressed in roots of a drought-tolerant genotype of *P. vulgaris*, the SSH technique was employed using cDNA synthesized from plants under severe drought stress of BAT 477 (tester) and Carioca 80SH (driver), harvested after 192 h of imposed stress.

A total of 1632 clones were randomly sequenced, generating 1572 valid reads that could be grouped into 189 contigs and 931 singletons, totalizing 1120 up-regulated unigenes for the BAT 477 genotype. Table 1 displays the most abundant contigs in numbers of reads annotated via BlastX and distributed according to biological process classes.

In a similar study, Rodrigues *et al*. (2012) [24] employed the SSH technique combined with high-throughput sequencing technology to investigate differentially expressed genes from two soybean cultivars, Embrapa 48 (tolerant) and BR16 (susceptible), under water deficit treatments. Twelve libraries were constructed using leaves and roots harvested in different stages of development, resulting in 6051 differentially regulated genes for Embrapa 48, and 4293 for BR16. As a basis for comparison, considering only the SSH libraries constructed for the tolerant genotype Embrapa 48, an average of 1008.5 unigenes were obtained in each of them, which leads us to believe that even though the traditional Sanger sequencing technique adopted in our work offers a short amount of reads per run, we were able to achieve a considerable high level of up-regulated unigenes.

**Figure 2:**
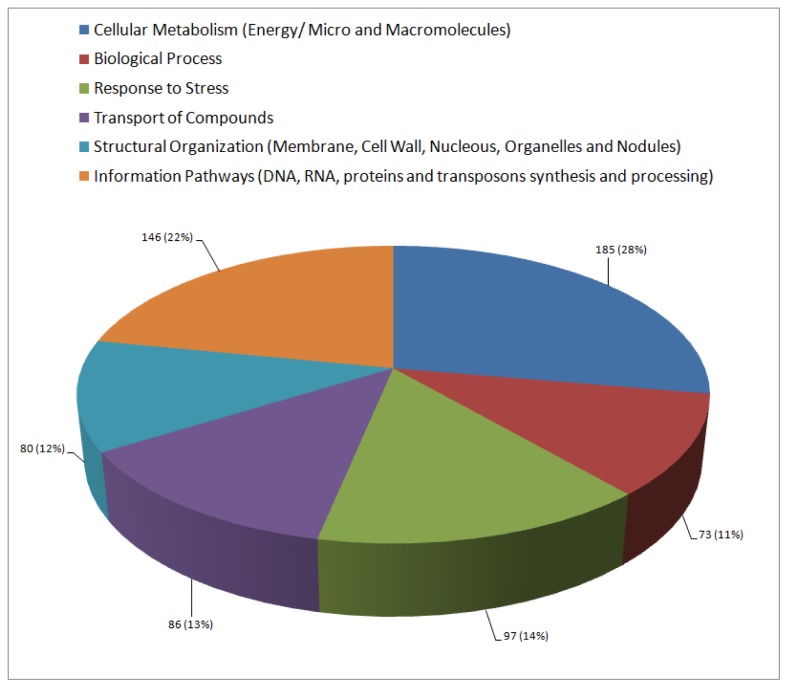
Functional classification of 667 ESTs (contigs and singlets) annotated as putative proteins. Each sector contains the number of ESTs per class and, in parentheses, the representation of each class in relation to the total amount of ESTs successfully annotated.

After BlastX search, 667 ESTs (59.5%) could be annotated as putative proteins, remaining 453 (40.5%) annotated as protein with unknown function or that had no homology to any sequence in the GenBank. Following that, a functional classification was carried out in order to better understand the biological processes that are enriched in the roots of the tolerant genotype of common bean in response to a drought stress. ESTs were classified into six distinct major functional classes as can be seen in Figure 2.

The most abundant functional class was “Cellular Metabolism” with 185 ESTs. This result was expected since plants that undergo water deficit are subjected to intense metabolic changes and suffer alterations in assimilation of carbon. These factors can lead to an integrated response at the whole plant level, which includes alteration in photoassimilates allocation at different plant organs and is a direct reflection of the reproductive ability [25]. Acclimatory changes in the root:shoot ratio or the temporary accumulation of reserves in the stem under water deficit are accompanied by alterations in carbon and nitrogen metabolism leading to a decay of reserves accumulation in the meristem [26].

Under water deficit, some legume plants like *P. vulgaris* also accumulate ureides in plant tissues, a metabolic reaction correlated to the inhibition of nitrogen fixation [27]. It was described in *P. vulgaris* that many of these compounds, mainly allantoate, accumulates in roots, shoots and leaves, but only a limited transient increase was observed in nodules from drought-stressed plants [27]. These results suggest that under drought, ureidic legumes metabolize these compounds, as part of a general response in which leaf senescence is triggered (probably with the involvement of ABA), to recycle enough nitrogen for the rapid generation of seeds. Further studies should be conducted in order to address the question of whether drought-tolerant or sensitive *P. vulgaris* cultivars do actually differ in their ureide content and perception to stress, thereby leading to differences in the induction of protective pathways, including the synthesis of ureides.

Regarding those sequences related to “Response to Stress”, 97 different ESTs were annotated, the third class being the most abundant. To better discuss this, we will present the main findings in the next section.

### 2.4. Major Response Mechanisms Activated

One of the groups of ESTs that most stood out in our library was related to the GTP-binding proteins (six different ESTs in total). These proteins are mainly regulated by G protein-coupled receptors (also found in our library; GenBank ID: |75749541|), a family of proteins involved in transmitting chemical signals into the cell [28], and act as a gateway to the triggering of all the signaling pathways that lead to cellular response to stress. They communicate signals from stimuli caused by the presence, for example, of hormones (ABA, ethylene, jasmonic acid), ions (Ca^2+^), or free radicals.

An important mechanism for maintaining cellular homeostasis during stress response is the detoxification system. During drought, although the absorption of ions by the cell via the plasma membrane sometimes offers the fastest and most efficient way for osmotic adjustment, in some cases, it may disrupt the metabolism. In situations of stress and osmotic imbalance, plants tend to accumulate Na^+^, partly due to a membrane potential that favors the passive influx of Na^+^ through channels and carriers, something that would be toxic to cells [2].

We have found an EST (GenBank ID: |75748673|) annotated as a putative membrane protein channel that coordinates the K^+^ efflux, the kefB. According to Bray *et al*. (2000) [2], this channel also has high affinity for Na^+^, but in high extracellular concentrations of this ion, the influx of K^+^ becomes blocked making Na^+^ lose affinity to the carrier and consequently inhibiting its influx, thus leading to a decrease of toxicity. In situations of extreme oxidative stress, the enzyme glutathione *S*-transferase, whose transcripts were also widely present in our library (GenBank IDs: |75749266|, |75749443|, |75748453|, |75748989|, |75749428|, |75749304|, |75749502| and, |75749502|), promotes the binding of glutathione to oxidized compounds such as lipids and ions that may be toxic to the cells. This enzyme provides the efflux of K^+^, leading us to believe that such a system increases the concentration of this ion in the extracellular environment and thereby reduces the influx of Na^+^.

Another system to prevent the accumulation of Na^+^ in the cytoplasm that was identified is the kidnapping of this ion by the cell vacuole. In plants with heavy accumulation of Na^+^, Na^+^/H^+^ vacuolar transporters (antiport system) are activated by a differential on chemical potential through the tonoplast by protein pumps called V-H(+)-ATPases (GenBank IDs: |75748882|, |75749301|, |75748847|, |75748861|), and promote such capture of Na^+^ excess.

When under water deficit, the osmotic balance of cells may also be regulated by the presence of transmembrane protein channels known as aquaporins that make both plasma and vacuolar membranes more permeable to water [29]. The levels of transcripts related to these proteins increase inside the cells as they gradually recover their turgor. In our library, we identified three ESTs annotated as aquaporins: one (GenBank ID: |75748779|) similar to aquaporin PIP2.1 of *Ricinus communis*, other (GenBank ID: |75748631|) similar to PIP2 of *G. max* and the third (GenBank ID: |75749342|) was a putative aquaporin identified previously in *P. vulgaris*.

We also detected sequences encoding for enzymes of synthesis and storage of compatible solutes that help maintain the osmotic gradient needed for cell turgor. According to Wang *et al*. (2003) [30], there are three major groups of osmolytes: amino acids (e.g., proline), quaternary amines (e.g., glycine betaine) and polyols/sugars (e.g., mannitol, trehalose).

In plants, proline is synthesized from glutamic acid via Delta(1)-pirrolyne-5-carboxylate by two enzymes P5C, a synthetase and a reductase [30]. These two intermediaries were identified in our SSH library, along with ESTs for proline-rich proteins (GenBank IDs: |75748690|, |75749470|, |75748843|, |75748573|, |75749321|, |75749128|), and for Delta(1)-pirrolyne-5-carboxylase proteins (GenBank IDs: |75748599|, |75748563| and |75749733|).

The biosynthesis pathway of trehalose was also identified as active by the presence of an EST coding for trehalose-6-phosphate synthase (GenBank ID: |75748369|). Other genes associated with the synthesis of complex carbohydrates were also found: sucrose synthase (GenBank ID: |75749186|), raffinose synthase (GenBank ID: |75749683|) and l-rhamnose synthase (GenBank ID: |75748387|), the latter being associated with the synthesis of an extracellular mucilage that may be involved in protection against desiccation [31].

As previously stated, one of the major consequences directly associated with water deficit stress is the release of ROS, which causes extensive cell damage and can lead to inhibition of photosynthesis [21]. In order to detect and eliminate these highly toxic compounds, the evolution has selected in plants a set of engines to search and dispose of these byproducts. The main transcripts of these pathways were found in our library: catalase (GenBank ID: |75748630|), superoxide dismutase (SOD) (GenBank IDs: |75748853|, |75749444|), ascorbate peroxidase (GenBank IDs: |75748953|, |75749654|), and other peroxidases (GenBank IDs: |75748731|, |75749130| |75748967|, |75749555|, |75749555|, |75748951|, |75749341|), and a thioredoxin peroxidase (GenBank ID: |75749664|).

Another pathway that signals for an antioxidant system is the MAPK module activated initially by a series of receptors/sensors like tyrosine kinase, which will enable a number of other signal-transducing kinases also known as mapks, that were found in our study (GenBank IDs: |75749581| and |75749350|, respectively).

Signaling pathways mediated by Ca^2+^ seem to be important for the process studied here once an intense presence of ESTs corresponding to Ca^2+^ carriers was observed. The concentration of this ion in the cytosol is low, and under stimulation, can be released from intracellular storages or be transferred inside via Ca^2+^ protein carriers [32]. The ESTs (GenBank IDs: |75748626|, |75749482|, |75748347|, |75748604|, |75749597|, |75748699|) correspond to different genes that encode distinct Ca^2+^ carriers still not explored. The contig 80 is the only one that differs from the others and is probably related to an antiport calcium:sodium system.

However, Ca^2+^ channels represent only one type of sensor for responses driven by stress signals. According to Dodd *et al*. (2010) [32], CDPKs (calcium-dependent protein kinases) are important sensors of Ca^2+^ influx in plants during stress signaling. CDPKs are serine/threonin protein kinases, such as those widely represented in our library (GenBank IDs: |75749220|, |75749362|, |75749581|, |75749425|, |75748620|), which have a *C*-terminal calmodulin domain (GenBank ID: |75749254|) with up to four EF-hand motifs that can directly bind to Ca^2+^.

The CDPK pathways also are predicted to be closely related to the increased expression of genes encoding LEA proteins [33]. The activation of LEA genes in fact represents a way of repairing the damage caused by the occurrence of stress [34]. These proteins are known to accumulate in plant cells that are under extreme desiccation conditions preserving proteins structure and the integrity of membrane proteins and acting in the sequestration of ions in stressed tissues [35]. We found 19 ESTs related to different groups of LEA proteins: nine corresponding to those similar to LEA5 from *G. max* (GenBank IDs:|75748394|, |75749216|, |75748401|, |75748773|, |75748546|, |75748890|, |75748291|, |75749560|, |75749523|), two corresponding to LEA3 from *P. vulgaris* (GenBank ID’s: |75749086|, |75748799|), two LEA3 similar to those in *G. max* (GenBank IDs: |75748444|, |75748410|), two 18-PvLEA from *P. vulgaris* (GenBank IDs: |75749723|, |75748622|), one similar to a LEA4 from *G. tomentella* (|75748634|) and three LEA3 similar to those in *Ammopiptanthus mongolicus* (GenBank IDs: |75748734|, |75749534|, |75749535|) that have a functional domain related to a LEA protein in group 2 (dehydrins).

Another important group of proteins often related to environmental stress responses are the transcription factors (TFs) [36] that can enable or disable the expression of a particular set of genes, thus activating or repressing specific or broad pathways related to drought tolerance in plants [37]. TFs may respond differently to each type of stimuli caused by stress; on the other hand, some stress-responsive genes may share the same TFs, as evidenced by the significant overlap of gene expression patterns that are induced in response to different stresses [38].

TFs can be classified into several families based on the structure of their binding domains [36]. From a number of TF families described, those that have already been implicated in the regulation of stress responses are: MYB, MYC, ERF, bZIP, and WRKY [36]. Table 2 lists the main transcription factors identified in our library grouped according to their families.

The best-studied group of TFs involved in abiotic stress tolerance comprises the DREB genes [39]. Many osmotic stress-inducible genes contain a conserved DRE (drought responsive element) in their promoters [40]. Several cDNAs encoding DRE binding proteins have been isolated and are shown to bind and activate genes containing DRE sequences [41]. On the other hand, NAC family represents one of the largest TF families in plants, being commonly related to lateral root growth and its activation mediated by auxin when under abiotic stress stimuli [42].

Another important EST found is related to a MYB transcription factor, often related to increasing stress-protective proteins and efficient stomatal closure under water-deficient conditions [36]. The bZIP family, that also had some representatives in our library, is involved in many regulatory and developmental processes, including ABA and stress signaling, seed maturation and flower development [43], playing also an important role in abiotic stresses responses [44].

This large and heterogeneous set of TFs that we have found suggests an intense regulation of gene expression in the roots of BAT 477 under drought stress, leading us to believe that further studies specifically focused on TFs would highlight the main molecular mechanisms used by this genotype to surpass this stress.

### 2.5. Validation of the SSH Library by RT-qPCR

The goals for this experiment were to answer two main questions. At first, it was necessary to analyze the expression pattern of all 10 selected genes in Carioca 80SH in order to check whether these genes were exclusive to the tolerant genotype (BAT 477), or if they were also expressed in the susceptible one. The second, and most important, compare the expression patterns between both cultivars under water deficit to confirm that they are higher in the tolerant genotype, validating our experiment.

**Figure 3:**
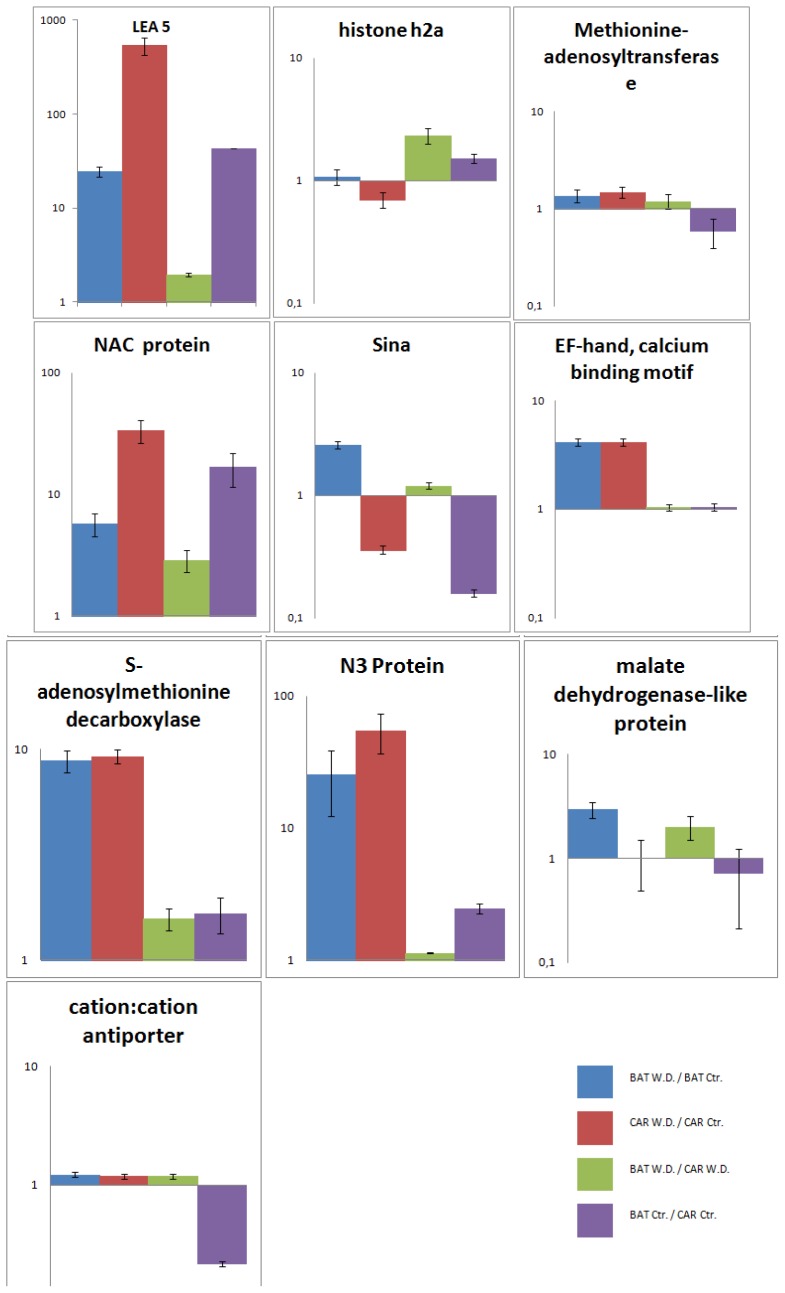
Relative Expression Levels of 10 transcripts selected for the validation assay. For each chart the columns are organized according to the following order: BAT W.D./BAT Ctr. (BAT 477 under water deficit relative to BAT 477 in control condition); CAR W.D./CAR Ctr. (Carioca 80SH under water deficit relative to Carioca 80SH in control condition); BAT W.D./CAR W.D. (BAT 477 under water deficit relative to Carioca 80SH under water deficit); BAT Ctr./CAR Ctr. (BAT 477 in control condition relative to Carioca 80SH in control condition).

Normalized expression values of samples were contrasted against each other and four Relative Expression data for each gene were plotted in the graphics presented on Figure 3.

In general, the 10 selected genes were more expressed in BAT 477 than in Carioca 80SH when they were submitted to water deficit, therefore validating our SSH library (Figure 3). Moreover, three expression patterns could be highlighted as predominant among our four comparisons.

The first pattern observed includes the genes LEA 5, NAC protein, N3 protein, EF-hand calcium binding motif, and *S*-adenosylmethionine decarboxylase. All of them were up-regulated during stress in both the genotypes; under the control condition we could observe that the basal expression levels of these genes are lower in Carioca 80SH; and comparing both under stress (BAT W.D./CAR W.D.) these differences get shorter (Figure 3). These observations show that, for these genes, although an increase in expression for BAT 477 occurs during drought, the most accentuated increase in expression occurs for Carioca 80SH. On the other hand, despite the fact that they are more abundant in the tolerant genotype, they have a greater regulation in the susceptible one, perhaps because they are more necessary to the susceptible one in order to surpass the drought-stress period than for the tolerant genotype.

The second gene expression pattern is related to three of the selected genes: methionine-adenosyltransferase, malate dehydrogenase-like protein and cation:cation antiporter (Figure 3). These genes are also activated in both genotypes submitted to water deficit. However, when we examine their basal expression levels in the control condition, we can see that it is lower in BAT 477, thereby indicating a faster activation of these genes in BAT 477, perhaps in order to plant survival during the stress period. The product of the malate dehydrogenase-like protein transcript is associated with basic metabolic processes, like the citric acid cycle, and the maintenance of its expression in Carioca 80SH was expected, in order to secure the survival of the plant, but the increase for BAT 477 may be related to an increase in metabolic pathways associated with the synthesis/degradation of basic compounds needed for providing energy to keep the stress response machinery up.

The third pattern observed is related to the genes Sina and Histone h2a. Both of them, as in the second pattern, have their basal expression levels lower in BAT 477 than in Carioca 80SH. However, while these genes are up-regulated in BAT 477 and submitted to stress, they are down-regulated in Carioca 80SH (Figure 3). Histone h2a protein have specialized roles in stabilization and folding of chromatin [45]. Chromosome condensation prior to cell division due to the presence of linker stones has recently being correlated to transcriptional repression of genes since transcriptional regulation is dependent also of the local and regional chromatin environment of genes [46]. In this case, the enrichment of this transcript in the tolerant genotype may be leading to the repression of specific metabolic pathways, therefore allowing the plant to save important cellular resources in order to maintain the basal metabolism regular during the stress period.

The seven in absentia (SINA) proteins are E3 ligases that often active as dimers and are highly associated with target proteins for ubiquitination [47]. They were described as having a role in nodulation, shoot elongation, leaf size, and lateral root (LR) number [48]. In our case, the plant may be up-regulating the expression of this factor in order to increase nodulation during the stress period, thus diminishing its LR formation. This may be a response to previous field observations correlating the superior drought-stress tolerance of the genotype BAT 477 due to its improved capacity of nodulating, and thus nitrogen fixation, in water defective soils [49]. For Carioca 80SH, our results indicate that it is more important to sustain its regular nodule formation rates under normal circumstances than for BAT 477; however, once under drought, this situation suffers an inversion. Therefore, for BAT 477, it is more likely a security system which is activated during stress periods in order to sustain nodulation rates at high levels and then keep plant homeostasis. While for Carioca 80SH, where the gene is repressed, the plant would be able not only to save important resources needed for survival during stress, but also to maintain plant growth, mainly LR growth.

### 2.6. Time Scale Gene Expression Analysis

In a second study, 31 different ESTs (related to genes commonly understood as important to drought-stress response in literature) were selected from the SSH library. This study was conducted using root samples obtained from the same experiment described earlier (BAT 477 and Carioca 80SH, treated/untreated) and considering different periods of harvesting: 72, 144 and 192 h after starting the stress and 24 h after plants were re-watered following 192 h of imposed stress.

**Figure 4:**
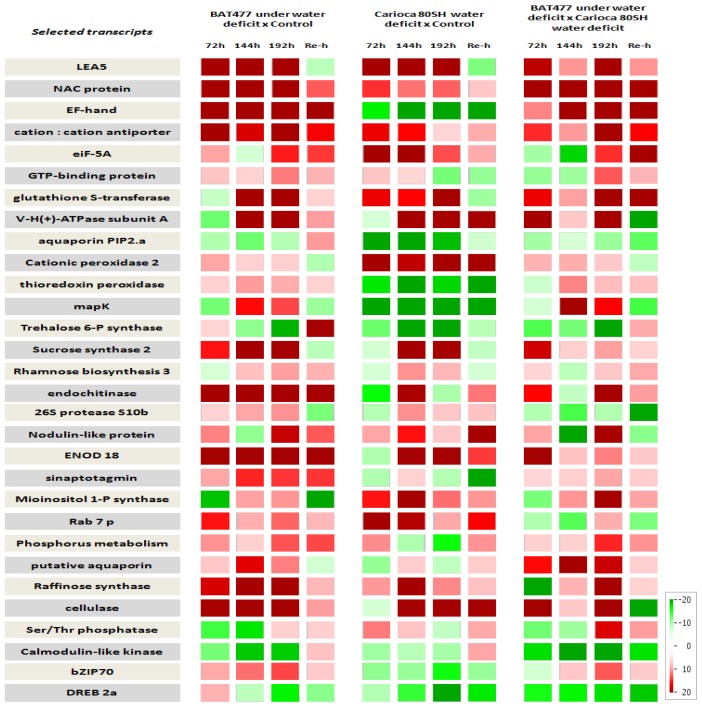
HeatMap containing the expression patterns of the 31 selected ESTs obtained by RT-qPCR. Gene expression patterns ranging from −20 (green color mostly down-regulated) to +20 (red color as mostly up-regulated). Relative Expression was achieved by contrasting different samples: Column 1 − BAT 477 treated/untreated; Column 2 − Carioca 80SH treated/untreated; Column 3 − BAT 477 treated/Carioca80SH treated.

By the HeatMap shown in Figure 4, we realized that the usually expected pattern of constant and progressive up-regulation of genes through time during a stress period is not the rule, but the exception. For BAT 477, only twelve of the genes revealed this pattern (LEA5, NAC domain protein, EF-hand calcium-binding motif, mapK, sucrose synthase, rhamnose biosynthesis enzyme 3, 26S protease s10b subunit, ENOD18 factor, sinaptotagmin, raffinose synthase and cellulase), and for Carioca 80SH, only six of them (LEA5, cationic peroxidase 2, rhamnose biosynthesis enzyme 3, sucrose synthase, ENOD18 factor and cellulase). Unlike the usual pattern that is observed, there is a fluctuation in regulation with the genes up and down-regulating in relation to control conditions during the period of imposed stress.

In general, almost all the transcripts analyzed were shown to be most up-regulated in the tolerant genotype (BAT 477), mainly after 192 h of stress as previously expected due to this being the time point used for SSH library construction. The genes that were most up-regulated in BAT 477 are: LEA5 (8.1-fold), NAC domain protein (30.9-fold), EF-hand, calcium-binding motif (26.8-fold), cation:cation antiporter (10.5-fold), and cellulase (7.75-fold) (Table S1).

Although these results were expected, we were able also to detect that some genes (calmodulin-like kinase, DREB2a, PIP2.a and trehalose 6-P synthase) showed a repression in their expression levels in BAT 477 after 192 h of stress. This decay in expression was also detected for the susceptible genotype, although in a higher intensity which can lead us to believe that the selection of these transcripts by the SSH technique was more due to its different abundance between genotypes in this particular moment than by their importance in conferring tolerance to BAT 477.

The analysis of relative expression data for the rehydration event (Re-h columns in Figure 4) proved to be very informative. Previously, it was thought that after a rehydration event following a drought period, the expression rates of these genes would diminish for both cultivars. Meanwhile, some genes revealed a differentiated expression pattern. For BAT 477, some genes that proved to be actually down-regulated during all the period analyzed (aquaporin PIP2.a, trehalose 6-P synthase and calmodulin like kinase) became suddenly up-regulated after rehydration; the same being observed for calmodulin like-kinase, Ser/Thr protein phosphatase, cation:cation antiporter and GTP-binding protein in Carioca 80SH, revealing it to be more important for re-acclimation and not for conferring drought tolerance. Other transcripts, like an EF-hand binding motif, endochitinase, rhamnose biosynthesis enzyme and ENOD18 factor in BAT 477, and V-H(+)-ATPase subunit and cationic peroxidases in Carioca 80SH, did not exhibit, or almost did not exhibit, shifts in their relative expression data during all the process, revealing not to be strongly correlated to the drought-stress response for these particular circumstances analyzed.

Whilst this second study has offered a set of candidate genes that may be vital for the drought-stress response in the tolerant genotype BAT 477, some of the genes were also revealed, at some point, to be up-regulated in the susceptible. We can see, however, in the third group of columns in Figure 4, that in lower levels, six of these genes (aquaporin PIP2.a, trehalose 6-P synthase, 26S proteinase S10b, Rab7p, DREB2a and calmodulin-like kinase) are more expressed in the susceptible genotype. These results prove that different genotypes can adopt and adapt different strategies during a stress event in order to develop tolerance to it. Furthermore, the main genes by which the tolerant genotype BAT 477 can mobilize the responses to drought are also present in Carioca 80SH. Although the second one is not able to use them in the same intensity, the development of strategies that would improve the expression of these candidate genes in the susceptible genotype would be of great interest in plant-breeding programs in order to obtain new cultivars more tolerant to drought.

### 2.7. Genevestigator Cluster Analysis

**Figure 5:**
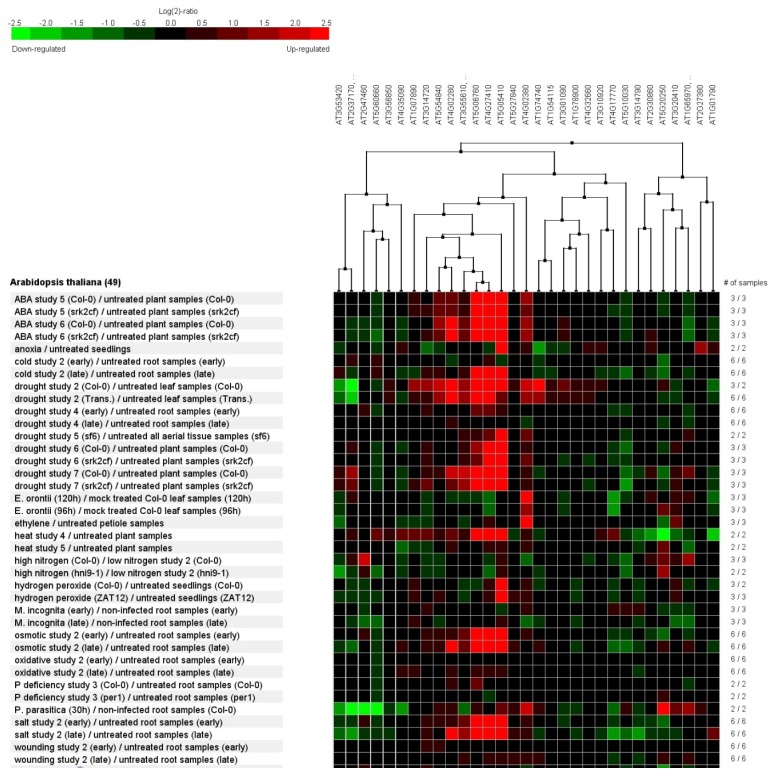
Clustering analysis conducted via Genevestigator software [50]. Microarray’s gene expression data from abiotic and biotic treatments and experiments were obtained from the plant model *Arabidopsis thaliana* and ATH1: 22 k array platform database. The list of the *Arabidopsis* orthologs definitions and the corresponding ESTs from our library are available in the Table S2. Gene expression patterns are represented in a log2 ratio ranging from −2.5 (green color mostly down-regulated) to +2.5 (red color as mostly up-regulated); *Arabidopsis* orthologs were hierarchically classified according to similar expression patterns across the selected dataset.

*A. thaliana* has one of the most comprehensively annotated genomes, which is supported by extensive experimental data. A clustering analysis was therefore conducted via Genevestigator software [50], analyzing the corresponding *Arabidopsis* orthologs of our main annotated ESTs (Table S2) in a series of experiments that collected several biotic and abiotic stress microarray assays using *Arabidopsis* roots as samples (Figure 5).

The Pearson’s correlation matrix obtained from the differential patterns in expression of the 31 selected transcripts (Figure 5) showed that five of them (Delta 1-Pyrroline-5-Carboxylate Synthase 2, Lea 4, LEA5, sucrose synthase and NAC protein) were not only up-regulated in *Arabidopsis* for all of the selected drought experiments (for whole plant samples and roots), but also for the other treatments, which is usually indicative of the same response patterns, such as ABA, cold, high salinity, and osmotic treatments.

The treatment that was shown to be most correlated with our work was the experiment of drought stress on leaves only. For this analysis, 18 of our selected transcripts were significantly up-regulated in leaves of *Arabidopsis*, and six were down-regulated. When we consider the drought-stress response in roots under drought and its control, only six transcripts were up-regulated (PIP 2, Delta 1-Pyrroline-5-Carboxylate Synthase 2, Lea 4 and NAC protein, map kinase and sucrose synthase), seven of them being down-regulated (putative aquaporin, LEA5, trehalose-6-phosphate synthase, OCS element binding factor 4, raffinose synthase, calmodulin-domain protein kinase and thioredoxin peroxidase). These divergences between our study and the patterns obtained for root samples in *Arabidopsis* may be related to the great differences in evolution and artificial selection of the two species, thus pointing to the relevance of whole transcriptome studies concerning species of agricultural interests.

Some of the analyzed transcripts (putative aquaporin, OCS element binding factor and raffinose synthase), were extensively down-regulated in *Arabidopsis* treatments and therefore must indicate interesting candidate genes for studies concerning acquired drought tolerance in *P. vulgaris*.

## 3. Experimental Section

### 3.1. Plant Material and Induction of Drought Response

Two common bean genotypes were used: BAT 477 which is an advanced line launched by CIAT (International Center for Tropical Agriculture) and selected for its growth and ability to fix nitrogen in symbiosis with *Rhizobium* in soils with P deficiency and water deficit conditions [48,49,51]; and Carioca 80SH that represents the most widely grown variety among farmers in Brazilian regions that are perpetually deprived of rain in growing seasons, leading to serious losses in production [52]. Due to its deprivation, Carioca 80SH was adopted as a model of inefficient genotype under drought in this work.

Seeds of BAT 477 and Carioca 80SH were superficially sterilized in a sodium hypochlorite solution in water (1:3) and transferred to germination papers. We defined four treatments for the greenhouse experiment, each containing five pots with one plant each: (i) BAT 447 under water deficit; (ii) BAT 477 with no stress (control); (iii) Carioca 80SH under water deficit; (iv) Carioca 80SH with no stress (control).

All treatments were kept under uniform irrigation conditions (300 mL of water per pot every two days) until plants reached the R5 phenological stage, then, all pots selected for “water deficit” treatment had their water supply interrupted. Moisture content was monitored daily by collecting samples of soil from each pot and drying them until constant weight. Mean moisture contents were determined for each treatment following the formula *u* (%) = *W*_w_/Rdw × 100, where *W*_w_ (“water weight”) is related to the difference between the wet weight of the sample and its dry weight, and Rdw (“real dry weight”) is the dry weight of each sample individually.

Sampling of plant material was carried out after 72, 144 and 192 h of drought induction. Leaves were collected for protein extraction and catalase ezymatic activity determination; roots were collected in order to obtain total RNA for the transcriptomic study. Biological samples from five plants per treatment were collected without washing, frozen in liquid Nitrogen and kept at −80 °C.

A parallel study was also conducted with the same genotypes, Carioca 80SH and BAT477, with the goal of obtaining the RWC (%) of leaves induced through the application of a 10% PEG solution during 6 h. Leaves were rapidly weighed after harvesting in order to obtain the “fresh weight” (*W*_f_); after this procedure, the leaves were drowned in distilled water for 4 h under room temperature and constant lightning. Then, leaves were weighed again in order to obtain the “turgid weight” (*W*_t_) and dried for 24 h at 40 °C to obtain the “dry weight” (*W*_d_). RWCs were calculated following the equation: RWC (%) = [(*W*_f_ − *W*_d_)/(*W*_t_ − *W*_d_)] × 100.

### 3.2. Catalase Enzymatic Activity Determination

Bulks composed of leaves from five plants collected from the experiment were used for total protein extraction. Extraction buffer (potassium phosphate pH 7.0, 1 mM EDTA, 3 mM DTT) and 1% PVPP (polyvinylpolypirrolidone) were added to 50 mg of sample. After centrifugation (4 °C, 15 min, 14,000 × *g*), the supernatant was transferred to a new tube. The extract was centrifuged again for 2 min for additional leaf tissue removal and the supernatant was stored in ultrafreezer (−80 °C).

Assays were performed according to [53]. To a total of 100 μL of each diluted protein sample, 3 mL of potassium phosphate buffer 50 mM pH 7.0 was added, followed by 60 μL of H_2_O_2_. Samples were read at 240 nm wavelength in a Nanodrop 2000^©^ spectrophotometer over 2 min, with readings taken every 10 s. The results were expressed in μmol/min/mg protein.

### 3.3. SSH Library Construction

For SSH library construction, we selected the samples collected after 192 h of suspension of irrigation from both cultivars because they exhibited relatively low soil moisture content average in their substrate, but still not enough for the plants to reach permanent wilting point [51].

Before RNA extraction, biological replicates for each treatment were ground in liquid Nitrogen and mixed together in order to obtain four bulks. Total RNA was extracted with Trizol (Trizol Reagent^®^ LS, Invitrogen, Carlsbad, SC, USA) following manufacturer’s recommendations. The RNA samples were quantified by spectrophotometry at 260 nm and 280 nm wavelengths using NanoDropTM 2000c, and RNA quality was verified by electrophoresis on a 1.6% agarose gel. mRNA of each sample were purified using the Dynabeads^®^ mRNA Purification Kit (Life Technologies, Carlsbad, SC, USA) according to the manufacturer’s instructions.

From the mRNA samples, we constructed two cDNA libraries, one for each genotype under water deficit, using the PCR cDNA Synthesis Kit SMARTer™ and Advantage^®^ 2 PCR Kit according to the manufacturer’s instructions (Clontech Laboratories, Inc., Montain View, CA, USA). BAT 477 cDNA library was used as the “tester,” and Carioca 80SH was used as the “driver”.

PCR products obtained at the end of the SSH library construction were loaded in agarose gel Low-melting-point 1.6% in 1× TSB buffer, and the smear pattern observed was excised and purified using the GFX™ PCR DNA and Gel Band Purification Kit (GE Healthcare, Buckinghamshire, UK). The fragments obtained were bonded to the pGEM^®^—T Easy Vector (Promega, Fitchburg, WI, USA) and cloned in *Escherichia coli* (strain DH5α) for sequencing. A total of 1632 clones were sequenced at the Center for Molecular Biology and Genetic Engineering of Unicamp (CBMEG, Campinas, SP, Brazil), using the BigDye Terminator v3.1 Kit (Foster City, CA, USA) and an automated DNA capillary sequencer (ABI PRISM^®^ 3700 DNA Analyzer (Applied Biosystems, Foster City, CA, USA).

### 3.4. Bioinformatics Analyses

Phred tool [54] was used to assign values to the chromatograms and ph2fasta [54] was adopted for the conversion of reads with phred values ≥ 20 into FASTA files. Vector and poli-A sequences were subsequently removed using Crossmatch [54], followed by low quality sequences removal using blastall [55], crossmatch [54], SWAT [54], and other programs that employ *ad hoc* pattern-matching written in Pearl. For data interpretation, it was applied Parsers software built in the Pearl language [56].

Resulting reads were grouped by phrap tool [54] with arguments fixed on predetermined values (penalty—15, bandwidth 14, minscore 100, shatter_greedy). Contigs were achieved with CAP3 [57].

Functional annotation of the ESTs was performed based on similarity with common bean sequences and other related species, available in public databases [55], using the BlastX tool. For this purpose, only redundant sequences with *E*-value ≤ 1 × 10^−5^ were annotated; in addition, other parameters such as greater significant alignment (“best hit”) measured by the score and personal criteria based on related published data of genes described as involved on plant drought-stress response were also used.

For functional classification of the transcripts the Combined Scheme (CS) model developed by [58] was elected. The only change that was made to the CS model was the addition of the functional classes related to responses to biotic and abiotic stresses that are present in the system of the MIPS Functional Catalogue Database [59].

As *Arabidopsis thaliana* has one of the most comprehensively annotated genomes, and in many cases is supported by extensive experimental data, after annotation and functional classification, 31 individual bean ESTs obtained in our library were selected for their abundance, as well by classification in important metabolic pathways related to stress signaling and response, and then were aligned (tBlastX) with *Arabidopsis* transcript sequences using the TAIR10 database [60]; for this purpose, only redundant sequences with *E*-value ≤ 1 × 10^−5^ were annotated.

The putative *Arabidopsis* gene orthologs (AGI numbers) obtained were used to identify probes designed for the ATH1: 22 k array *Arabidopsis* platform and then accessed under different stress conditions by using Genevestigator clustering analysis [50].

### 3.5. RT-qPCR for SSH Library Validation and Time Scale Gene Expression Analysis

Total RNA extraction procedure was conducted in the aforementioned manner by using the root samples of BAT 477 and Carioca 80SH in both their drought-stressed and control conditions.

Primers were designed using Primer3 program [61] and their quality checked with NetPrimer[62]. “Table S3” shows the specifications of the 10 optimized primers used for the validation experiment and the 31 optimized primers used for the time scale gene expression analysis.

RT-qPCR was carried out in two steps. cDNA synthesis was obtained with Maxima™ First Strand cDNA Synthesis Kit (Fermentas, Waltham, MA, USA) in the following reaction: 2 μL of 5× Reaction Mix, 1 μL of Max Enzyme; 1 μL of RNA (100 ng/μL) and 6 μL of DEPC-water, which was incubated at 25 °C for 10 min, 50 °C for 15 min and 85 °C for 5 min. Quantitative PCR reactions were carried out using the Maxima SYBR Green qPCR Master Mix Kit (Fermentas, Waltham, MA, USA) in the StepOnePlus™ RealTime PCR System (Applied Biosystems, Foster City, CA, USA) equipment. The reactions contained 1 μL of cDNA, 5 μL of SYBR Green 2X, 1 μL of forward and reverse primers (2.5 pmol each) and 2 μL of DEPC-water. Amplification conditions were as follows: 10 min at 95 °C; 40 cycles of 15 s at 95 °C, 20 s at 59 °C and 20 s at 72 °C (with fluorescence data collection); and 95 °C for 15 s, 59 °C for 1 min and 95 °C for 15 s with data collection at each 0.7 °C increase in temperature to obtain melting curve data.

All samples were amplified in triplicate in order to quantify transcript levels of the target genes and two reference genes (act and skip2) [63]. After qPCR reactions, raw amplification data was exported to Excel spreadsheets and analyzed in the LinRegPCR 11.x software [64] to determine amplicon efficiency (*E*) and starting concentration (*N*_0_) values for each transcript. After normalization to the reference genes, relative expression ratios were calculated between contrasting samples.

From the Relative Expression data obtained for the time scale experiment, HeatMaps were constructed using the software MapMan 3.5.1R2 [65]. As the software does not have gene expression data for metabolic process mapped from *P. vulgaris*, the AGI numbers (Table S2) that were obtained as described in the previous section were used. These were then used as “access codes” to map our ESTs to the identifiers in the Affimetrix Ath_AFFY_ATH1_TAIR9_Jan2010 platform according to their cellular functions and then to visualize expression data in PageMan tull (inside MapMan 3.5.1R2).

## 4. Conclusions

A new set of ESTs from the root system of a drought-tolerant common bean (BAT 477) was obtained. Analyzed together, they revealed the potential metabolic pathways activated by these plants in order to acquire tolerance. Moreover, temporal gene expression analyses showed that, despite some transcripts having also been detected in the susceptible genotype (Carioca 80 SH), their regulation differs substantially between genotypes, thereby indicating molecular mechanisms as a target for breeding programs or for genetic engineering to create new cultivars with improved drought tolerance.

## Figures and Tables

**Table 1:** List of the most abundant contigs containing original access code in our library; number of reads in each contig; GI of the homologous sequences at NCBI (annotation); description of the EST and the correspondent species; and *E*-value. They are presented according to their functional classes.

Access code in library	Number of reads	GI number	Description/species	*E*-value
**Cellular metabolism (Energy/micro and macromolecules)**				

Contig147	3	|255579310|	pyruvate decarboxylase, putative (*Ricinus communis*)	4 × 10^−80^
Contig7	3	|83283965|	malate dehydrogenase-like protein (*Solanum tuberosum*)	1 × 10^−171^
Contig23	3	|255638912|	glyceraldehyde-3-phosphate dehydrogenase (*Glycine max*)	1 × 10^−119^
Contig28	3	|255540625|	glutaredoxin-1, grx1, putative (*Ricinus communis*)	2 × 10^−40^

**Biological processes**				

Contig123	3	|224094081|	spliceosomal complex, (*Populus trichocarpa*)	3 × 10^−35^
Contig171	3	|75304713|	Methionine adenosyltransferase, (*Phaseolus lunatus*)	1 × 10^−83^
Contig79	4	|156181612|	*S*-adenosylmethionine decarboxylase (*Phaseolus vulgaris*)	3 × 10^−25^
Contig127	4	|75304713|	Methionine adenosyltransferase, (*Phaseolus lunatus*)	5 × 10^−90^

**Abiotic stress response**				

Contig74	4	|42571665|	interferon-related developmental regulator family protein (*Arabidopsis thaliana*)	6 × 10^−53^
Contig105	3	|192910730|	light-inducible protein ATLS1, (*Elaeis guineensis*)	2 × 10^−30^
Contig14	3	|75708857|	group 3 late embryogenesis abundant protein, (*Phaseolus vulgaris*)	6 × 10^−23^
Contig61	3	|806310|	proline-rich protein, (*Glycine max*)	7 × 10^−18^
Contig37	4	|1732556|	LEA5 (*Glycine max*)	3 × 10^−34^
Contig97	4	|1350522|	LEA protein (*Picea glauca*)	3 × 10^−27^
Contig24	9	|1732556|	LEA5 (*Glycine max*)	3 × 10^−34^

**Biotic stress response**				

Contig3	3	|184202203|	isoflavone synthase 1 (*Vigna unguiculata*)	1 × 10^−85^
Contig3	3	|184202203|	isoflavone synthase 1 (*Vigna unguiculata*)	1 × 10^−85^
Contig17	9	|130835|	PvPR2 (*Phaseolus vulgaris*)	1 × 10^−79^

**Transport**				

Contig164	3	|61651606|	plastidic phosphate translocator-like protein1 (*Mesembryanthemum crystallinum*)	1 × 10^−61^
Contig80	4	|255587991|	cation:cation antiporter (*Ricinus communis*)	1 × 10^−39^
Contig2	3	|255552798|	ATP binding protein, putative (*Ricinus communis*)	8 × 10^−30^
Contig64	4	|255637247|	calcium ion binding (*Glycine max*)	2 × 10^−38^

**Structural organization (Membrane, cell wall, nucleus, nodulation and organelle)**				

Contig142	3	|255549412|	Vesicle-associated membrane protein, putative (*Ricinus communis*)	8 × 10^−31^
Contig137	3	|146233385|	abscisic acid ABA receptor (*Populus trichocarpa*)	1 × 10^−24^
Contig148	3	|194466205|	putative L24 ribosomal protein (*Arachis hypogaea*)	2 × 10^−23^
Contig11	5	|255584772|	histone h2a, putative (*Ricinus communis*)	2 × 10^−27^
Contig19	3	|57013900|	NitaMp027 (*Nicotiana tabacum*)	6 × 10^−33^
Contig83	4	|30682545|	ARF3 (ADP-Ribosylation factor 3) (*Arabidopsis thaliana*)	1 × 10^−59^

**Information pathways (processing of DNA, RNA and proteins/transposons)**				

Contig154	3	|187940303|	NAC domain protein (*Glycine max*)	8 × 10^−84^
Contig51	4	|20138704|	eIF-5A (*Manihot esculenta*)	7 × 10^−40^
Contig52	4	|255646048|	transferase activity (*Glycine max*)	2 × 10^−58^
Contig162	3	|155212489|	N3 protein (*Glycine max*)	1 × 10^−47^

**Unclassified**				

Contig72	3	|255626205|	unknown (*Glycine max*)	3 × 10^−78^
Contig87	3	|255639776|	unknown (*Glycine max*)	3 × 10^−71^
Contig98	3	|255647862|	unknown (*Glycine max*)	8 × 10^−55^
Contig145	3	|255646578|	unknown (*Glycine max*)	5 × 10^−47^
Contig6	4	|224101339|	predicted protein (*Populus trichocarpa*)	5 × 10^−30^
Contig64	4	|255637247|	unknown (*Glycine max*)	2 × 10^−38^
Contig77	4	|255637264|	unknown (*Glycine max*)	2 × 10^−10^
Contig82	6	|255629893|	unknown (*Glycine max*)	7 × 10^−27^

**Table 2:** Main transcription factors found in this study grouped according to their respective families. The list contains: GenBank IDs of our ESTs; GI, putative description, and correlated species used for annotation after BlastX search.

Transcription factor family	GenBank ID	GI/description/species
DREB	|75749717|	|292630743| Fe-S cluster assembly protein DRE2 homolog (*Glycine max*)
	|75748469|	|32480821| DREB (*Glycine max*)

ERF	|75749028|	|190361165| ethylene-responsive element binding factor 4 (*Glycine max*)
	|75749407|	|18643339| transcription factor EIL2 (*Vigna radiate*)

bHLH	|75749257|	|66947630| coiled-coil-helix-coiled-coil-helix domain containing protein (*Medicago truncatula*)

bZIP	|75749123|	|145652341| transcription factor bZIP70 (*Glycine max*)
	|75748580|	|223452524| leucine-rich repeat protein (*Glycine max*)
	|75748298|	|15148922| TGA-type basic leucine zipper protein (*Phaseolus vulgaris*)
	|75748883|	|255558466| F-box/LRR-repeat protein, putative (*Ricinus communis*)

NAC	|75749297|	|224088037| NAC domain protein, IPR003441 (*Populus trichocarpa*)
	|75748424|	|62546189| NAC4 protein (*Glycine max*)
	|75749318|	|224088037| NAC domain protein (*Populus trichocarpa*)
	|75748418|	|187940303| NAC domain protein (*Glycine max*)

MYB	|75748729|	|110931684| MYB transcription factor MYB185 (*Glycine max*)

GATA—factors	|75748743|	|255572876| GATA transcription factor, putative (*Ricinus communis*)

WRKY family	|75748775|	|151934195| WRKY36 (*Glycine max*)

Ubiquitous factors TFIIA e Sp1	|75748702|	|255566898| transcription initiation factor ia, putative (*Ricinus communis*)

IAA (auxin-responsive)	|75748737|	|255552973| Auxin-responsive protein IAA1, putative (*Ricinus communis*)
	|75748789|	|15226425| auxin-responsive family protein (*Arabidopsis thaliana*)

GRAS	|75748648|	|224106445| GRAS family transcription factor (*Populus trichocarpa*)
	|75749650|	|224106445| GRAS family transcription factor (*Populus trichocarpa*)

Heteromeric Factors	|75748712|	|193237557| transcription factor CCAAT (*Lotus japonicas*)

eIF2—alpha family	|75748325|	|255544025| translation initiation factor eif-2b (*Ricinus communis*)
	|75748617|	|20138704| Eukaryotic translation initiation factor 5A (*Manihot esculenta*)

Zinc finger	|75749674|	|161087182| C2-H2 zinc finger protein (*Glycine max*)
